# Hyperbaric Oxygen Therapy for Sensorineural Hearing Loss from *Streptococcus pneumoniae* Meningitis: A Case Report

**DOI:** 10.1055/s-0046-1816556

**Published:** 2026-02-18

**Authors:** Mohammed Abdalkarim, Merrick J. Harris, Summer M. Drees, Thaer Alhroob, Nahush Bansal

**Affiliations:** 1 Department of Medicine, The University of Toledo College of Medicine and Life Sciences, Toledo, Ohio, United States

**Keywords:** sensorineural hearing loss, meningitis, *Streptococcus pneumoniae*, hyperbaric oxygen therapy, audiogram

## Abstract

Sudden bilateral sensorineural hearing loss (SNHL) is a rare but debilitating complication of bacterial meningitis, which is most commonly caused by *Streptococcus pneumoniae* . While corticosteroids are a standard intervention to reduce inflammation and protect auditory function, adjunctive hyperbaric oxygen therapy (HBOT) may offer additional benefit, particularly in steroid-refractory cases. We report the case of a 57-year-old woman who developed profound bilateral SNHL following *S. pneumoniae* meningitis originating from a lumbar epidural abscess. After initiating HBOT, she experienced significant subjective and audiometric improvement in hearing. This case emphasizes the importance of early recognition, multidisciplinary care, and the potential utility of HBOT in managing meningitis-related hearing loss.

## Introduction

Bacterial meningitis is a life-threatening central nervous system infection with potential for devastating neurological sequelae. *Streptococcus pneumoniae* is the most frequent pathogen in adult cases. 1 One of the most feared complications is sensorineural hearing loss (SNHL), which can occur within hours of infection onset. 2 3

The pathophysiology of meningitis-related SNHL includes inflammation of the cochlea and auditory nerve, ischemic damage to inner ear structures, and direct cytotoxic effects of bacterial endotoxins. 3 4 Corticosteroids are the mainstay of therapy to reduce intracochlear inflammation. 5 However, in cases where hearing loss persists, hyperbaric oxygen therapy (HBOT) has emerged as a potential adjunctive treatment. 6 7

Herein, we present a unique case of bilateral SNHL in a patient with *S. pneumoniae* meningitis secondary to a lumbar epidural abscess, in which the patient experienced substantial improvement in hearing following HBOT.

## Case Report

A 57-year-old woman with a past medical history of coronary artery disease, hypertension, and sciatica presented to the emergency department with sudden-onset bilateral hearing loss, low back pain radiating to the left leg, headache, and nuchal rigidity. The patient had recently completed a 5-day course of prednisone 40 mg 2 days prior to presentation, prescribed for sciatic back pain. She denied trauma, fever, or recent infections. Neurological examination revealed neck stiffness and positive Kernig and Brudzinski signs. Otoscopic examination revealed no abnormalities with a normal external ear, normal ear canal, and normal tympanic membranes without bulging or erythema bilaterally. She was alert but unable to hear spoken words, requiring written communication.

Subsequent laboratory workup revealed leukocytosis (21.4), and two out of two blood cultures were positive for *S. pneumoniae* . Magnetic resonance imaging of the lumbar spine revealed a posterior epidural abscess from L2 to L4 with severe thecal sac compression. Lumbar puncture was contraindicated and not performed due to the location of this abscess and risk of spreading infection. The patient developed a fever of 38.7°C on hospital day 1. She was empirically started on intravenous ceftriaxone, ampicillin, and vancomycin. A bilateral L2, L3, and L4 laminectomy was performed for evacuation of the epidural abscess on hospital day 2. Cultures from the abscess contents sampled intraoperatively confirmed *S. pneumoniae* , and antibiotic therapy was deescalated and targeted based on organism sensitivity using ceftriaxone 2 g every 12 hours for 6 weeks and rifampin 300 mg twice a day for synergistic effect. Repeat blood cultures showed no growth after hospital day 2.

**Fig. 1:**
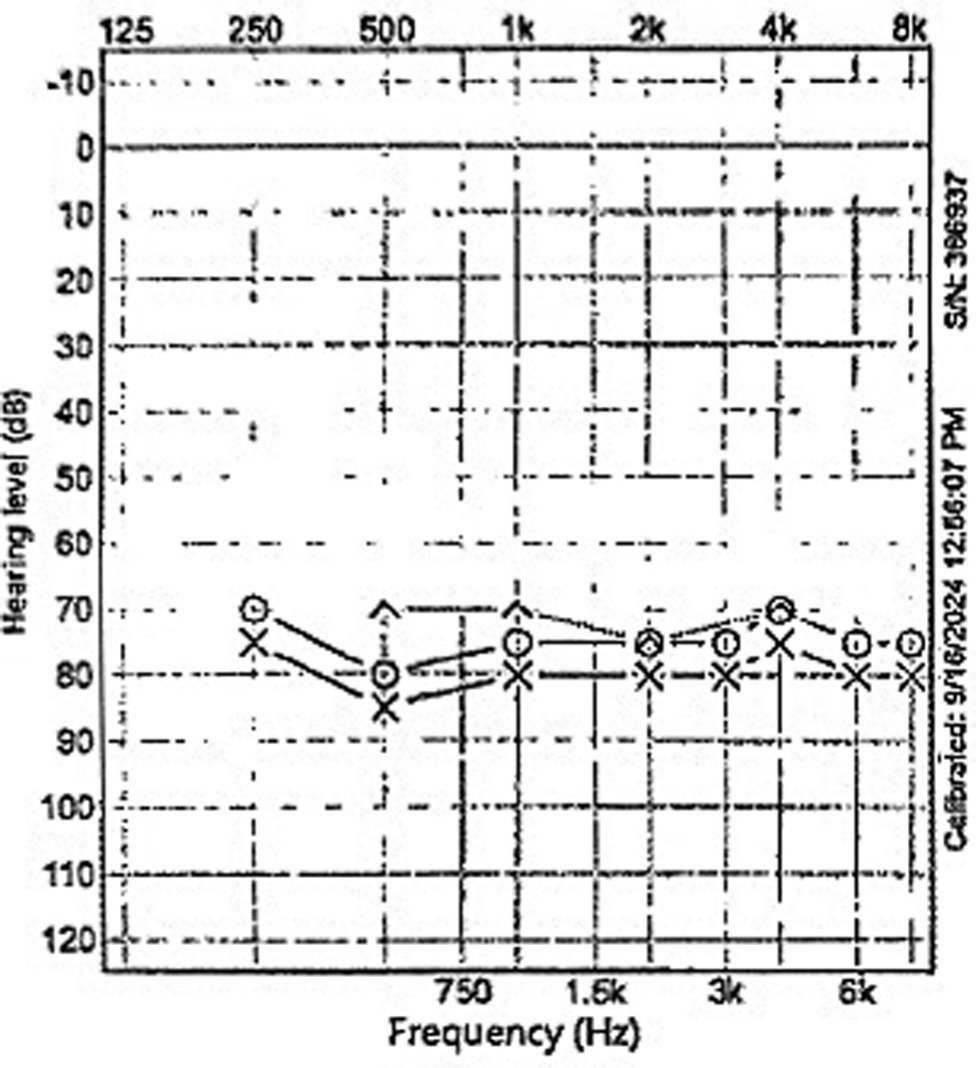
Initial audiogram, showed 20 and 32% speech discrimination in the right and left ears, respectively, and a speech detection threshold of 70 decibels hearing level (dBHL) in the right ear and 75 dBHL in the left ear with a sensorineural hearing loss pattern.

An audiogram performed on day 2 of hospitalization demonstrated severe bilateral SNHL with speech discrimination scores of 20% on the right and 32% on the left, as shown in Fig. 1 . Speech detection thresholds were 70 decibels hearing level (dBHL) on the right and 75 dBHL on the left, and pure-tone averages (PTAs) for air conduction of the right and left ears were 77 and 82 dBHL, respectively. Intravenous dexamethasone of 10 mg every 6 hours was started. On hospital day 6, the patient was switched to a 14-day course of prednisone 60 mg (1 mg/kg/day) followed by a 7-day taper.

**Fig. 2:**
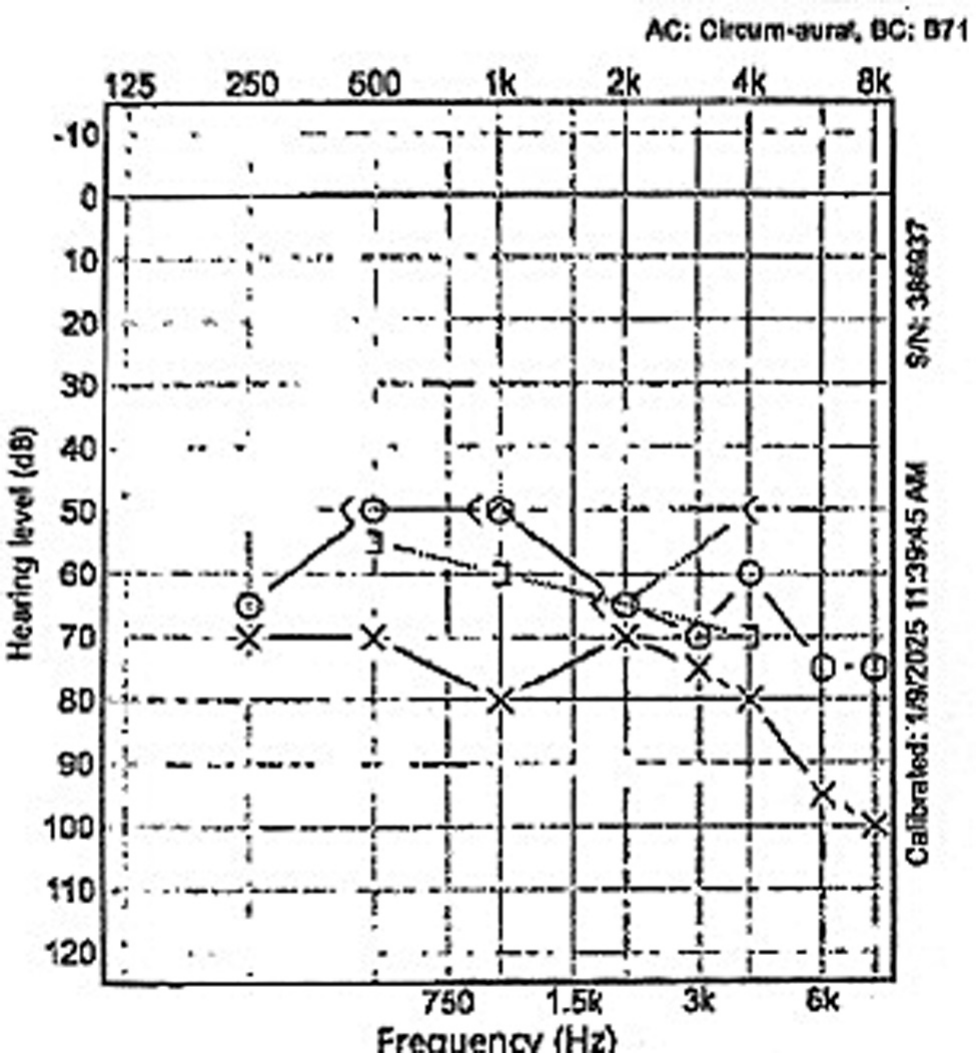
Repeat audiogram, 3 weeks later, speech discrimination was found to be 60% in the right ear and 40% in the left ear. Subjective improvement in hearing was also noted by the patient.

On hospital day 4, with no improvement in hearing after receiving high-dose intravenous systemic steroids, HBOT was initiated. The first session of hyperbaric oxygen was not tolerated by the patient due to right ear pain. On hospital day 6, bilateral myringotomy with tympanostomy tube insertion was performed to improve tolerability of HBOT by allowing equalization of pressures. This also facilitated the delivery of high-dose intratympanic steroids, thus Ciprodex drops were started and given twice daily for 10 days. After bilateral tympanostomy tube placement, the patient was able to tolerate HBOT without pain. HBOT was delivered in a monoplace chamber with the patient breathing 100% oxygen at 2.0 atmosphere absolute. Sessions were performed once daily, 5 days per week, for a total of 15 treatments over 3 weeks. Each session consisted of approximately 10 minutes of compression, 70 minutes at treatment pressure, and 10 minutes of decompression, following our institutional safety protocol for barotrauma mitigation. The first HBOT attempt prior to tympanostomy tube placement was aborted due to right ear pain consistent with middle ear barotrauma; no further barotrauma, oxygen-toxicity events, seizures, or significant claustrophobia were observed after tube placement. By the third session, the patient reported subjective improvement in hearing. A repeat audiogram after 3 weeks, shown in Fig. 2 , revealed speech discrimination of 60% on the right and 40% on the left, with improved speech detection thresholds bilaterally. PTA values for air conduction of the right and left ears measured at 55 and 73 dBHL, respectively. Clinically, the patient demonstrated meaningful improvement in communication ability.

## Discussion

Hearing loss following *S. pneumoniae* meningitis can occur early in the disease course and often results in permanent deficits in the absence of timely intervention. The bilateral and sudden-onset nature of the SNHL in this case underscores the aggressive nature of the infectious and inflammatory etiology leading to this complication.

Current standard treatment includes early administration of corticosteroids to reduce cochlear inflammation, which may preserve auditory function. 5 8 However, when hearing does not improve with medical therapy alone, alternative options are limited. HBOT offers a promising adjunct by increasing oxygen delivery to ischemic cochlear tissues, reducing edema, modulating inflammation, and promoting angiogenesis. 6 9

Several studies and case reports have suggested HBOT can improve outcomes in patients with idiopathic sudden SNHL including randomized trials and meta-analyses demonstrating greater absolute hearing gains when HBOT is added to steroid therapy compared with medical therapy alone. 6 7 10 More limited data exists for meningitis-related SNHL. Recent case reports of *S. pneumonia* meningitis complicated by bilateral SNHL describe partial hearing recovery in the better-hearing ear after combined systemic steroids and HBOT, while the contralateral ear remained profoundly impaired, suggesting that HBOT may confer ear-specific and incomplete benefit in this context. 11 12 These observations, together with our case, support considering HBOT as an adjunctive option when meningitis-related SNHL persists despite standard antibiotic and steroid therapy.

In our patient, HBOT appeared to partly reverse the damage incurred by the inflammatory and hypoxic insults to the inner ear due to meningitis. Audiometric and subjective improvements were observed after 3 weeks of therapy. PTAs, which represent an estimate of hearing ability by averaging hearing thresholds at different frequencies, improved by 22 dBHL in the right ear (77 to 55 dBHL) and by 9 dBHL in the left ear (82 to 73 dBHL). While causality cannot be definitively established, the initial resistance of response to steroid therapy and subsequent timely response to HBOT may indicate a therapeutic role in the treatment of meningitis-associated SNHL.

The main limitation of this case is the inability to assess the durability of hearing improvement, as audiometric follow-up was limited to the initial 3-week treatment period. Without long-term data, it is unclear whether recovery was sustained, progressed, or later declined. Concurrent interventions—systemic steroids, tympanostomy tubes, and intratympanic therapy—further limit evaluation of lasting benefit attributable to HBOT. Larger studies with standardized long-term follow-up are needed to clarify the persistent effectiveness of HBOT in meningitis-related SNHL.

Further studies are needed to define optimal patient selection, timing, and duration of HBOT in meningitis-related SNHL. Nevertheless, this case adds to a growing body of evidence supporting the early inclusion of HBOT in a multidisciplinary treatment plan when traditional therapies fail.

## Conclusion

This case report highlights the potential role of HBOT in the management of sudden bilateral SNHL caused by *S. pneumoniae* meningitis. In patients with refractory hearing loss despite appropriate antibiotic and steroid therapy, HBOT may offer a safe and effective adjunctive treatment to improve auditory outcomes. Prompt recognition and a collaborative, multimodal approach are crucial to mitigating long-term neurologic complications of meningitis.
